# Beyond Il-5: Metabolic Reprogramming and Stromal Support Are Prerequisite for Generation and Survival of Long-Lived Eosinophil

**DOI:** 10.3390/cells10040815

**Published:** 2021-04-06

**Authors:** Mackenzie E. Coden, Matthew T. Walker, Brian M. Jeong, Andrew R. Connelly, Reina Nagasaka, Sergejs Berdnikovs

**Affiliations:** Division of Allergy and Immunology, Department of Medicine, Northwestern University Feinberg School of Medicine, Chicago, IL 60611, USA; mackenzie.coden@gmail.com (M.E.C.); walkerm01@uchicago.edu (M.T.W.); brian.jeong@northwestern.edu (B.M.J.); andrew.connelly@northwestern.edu (A.R.C.); reina.nagasaka@northwestern.edu (R.N.)

**Keywords:** eosinophils, metabolism, bone marrow, glucose, carbohydrates, amino acids, stromal cells, chemotaxis, IL-5, biologics

## Abstract

Eosinophils play surprisingly diverse roles in health and disease. Accordingly, we have now begun to appreciate the scope of the functional and phenotypic heterogeneity and plasticity of these cells. Along with tissue-recruited subsets during inflammation, there are tissue resident eosinophil phenotypes with potentially longer life spans and less dependency on IL-5 for survival. Current models to study murine eosinophils ex vivo rely on IL-5-sustained expansion of eosinophils from bone marrow hematopoietic progenitors. Although it does generate eosinophils (bmEos) in high purity, such systems are short-lived (14 days on average) and depend on IL-5. In this report, we present a novel method of differentiating large numbers of pure bone marrow-derived eosinophils with a long-lived phenotype (llEos) (40 days on average) that require IL-5 for initial differentiation, but not for subsequent survival. We identified two key factors in the development of llEos: metabolic adaptation and reprogramming induced by suppressed nutrient intake during active differentiation (from Day 7 of culture), and interaction with IL-5-primed stromal cells for the remainder of the protocol. This regimen results in a higher yield and viability of mature eosinophils. Phenotypically, llEos develop as Siglec-F(+)Ly6G(+) cells transitioning to Siglec-F(+) only, and exhibit typical eosinophil features with red eosin granular staining, as well as the ability to chemotax to eotaxin Ccl11 and process fibrinogen. This culture system requires less reagent input and allows us to study eosinophils long-term, which is a significant improvement over IL-5-driven differentiation protocols. Moreover, it provides important insights into factors governing eosinophil plasticity and the ability to assume long-lived IL-5-independent phenotypes.

## 1. Introduction

Eosinophils are granulocytes that are found as relatively rare populations in multiple organs at homeostatic baseline; however, they significantly expand in tissues during morphogenetic and Type 2 immune responses [1]. Leukocytes can be generated via in situ hematopoiesis locally in tissues [2], although the majority of eosinophils are believed to originate in the bone marrow before being recruited to inflammatory sites [3,4,5]. Eosinophils play surprisingly diverse roles in health and disease, such as during mammary gland development [6], type 2 allergic diseases, wound healing [7,8], helminth defense [9], and cancer [10]. Recently, more information has come to light in regard to the surprising heterogeneity and plasticity of eosinophil granulocytes [1]. Along with inflammatory eosinophils in activated state, there are tissue resident subsets of these cells, which are likely to have longer life spans and differential sensitivity to cytokines [11,12,13]. Interestingly, it has been shown that such tissue resident eosinophils are not dependent on IL-5 for their survival [11], which is in line with research showing that IL-5 is necessary for differentiation of eosinophil granulocytes but dispensable for their maintenance [14]. Other factors, including tissue stroma and extracellular matrix (ECM) [15,16], or cytokines such as granulocyte-macrophage colony-stimulating factor (GM-CSF) or IL-3, could maintain such populations in tissues [14]. Given the growing interest in applications of biologics to target eosinophils via IL-5 and IL-5Rα axis in allergic and other inflammatory diseases (mepolizumab, benralizumab) [17,18,19], it is relevant to examine factors sustaining eosinophils in alternatively activated, long-lived and IL-5-independent states. Given they have diverse roles in health and disease, it is also equally important to develop the necessary tools for studying the phenotypic and functional heterogeneity of these cells.

Excellent protocols were previously developed for generating good numbers of eosinophils from murine bone barrow hematopoietic progenitors. Currently, the most accepted murine eosinophil differentiation protocol is one published by Dyer et al. [20], which involves a two-week cytokine treatment regimen using stem cell factor (SCF), FMS-like tyrosine kinase 3 ligand (FLT3 ligand), and IL-5. This results in large numbers of differentiated eosinophils at high purity [20]. This protocol is an improved version of another, earlier culture technique by Ishihara et al. [21], where murine bone marrow cultured in IL-5 alone resulted in pure eosinophil cultures, but at significantly lower numbers. Though both of these protocols differentiate eosinophils at high purity, they have common shortcomings. First, both protocols involve frequent addition of cytokine, which becomes costly. Second, once eosinophils reach maturity, they only remain viable for a few days, making for short time-windows in which experimentation must take place. Third, the resulting eosinophils are maintained and activated artificially by constant treatment with IL-5, which does not accurately represent homeostatic or long-lived subsets of eosinophils.

To address some of these challenges, we developed a novel bone-marrow-derived eosinophil culture protocol that extends longevity of these cells. It uses a short time frame of cytokine treatment (14 days) as a differentiation step, but consequently requires no further IL-5 treatment to produce eosinophils with high purity in large numbers. Remarkably, they survive up to 40 days in culture. The resulting phenotype is more reflective of “resident” cells typically found in homeostatic tissues. These eosinophils, which we will refer to as “llEos” (“long-lived eosinophils”) require stromal interaction and metabolic reprograming during their differentiation, which allows them to live longer than those cultured under any other method. In this manuscript, we detail the culture conditions necessary to sustain the long-lived phenotype, and provide further phenotypic and functional characterization of these cells.

## 2. Material and Methods

### 2.1. Mice and Bone Marrow Extraction

Murine bone marrow was harvested from 10–12-week-old wild-type BALB/c mice (the Jackson Laboratory, Bar Harbor, ME, USA) by flushing femurs and tibiae with RPMI 1640. Cells were cultured in complete media containing RPMI 1640 with l-glutamine, 1% penicillin/streptomycin, 1% l-glutamine, 1% MEM non-essential amino acids, 1% sodium pyruvate, 2.5% HEPES, 20% heat-inactivated FBS, and 0.0000035% 2ME. All animal experiments were approved by Northwestern University’s Institutional Animal Care and Use Committee (IACUC). All methods involving mice were performed in accordance with relevant guidelines and regulations.

### 2.2. Conventional Bone Marrow-Derived Eosinophil (bmEos) Culture

Eosinophils were cultured according to a published protocol by Dyer et al. [20]. In brief, cell media was supplemented with murine SCF (Peprotech, Cranbury, NJ, USA; 100 ng/mL) and FLT3-L (Peprotech, 100 ng/mL) from Days 0 to 4. On Day 4, cells were given fresh media containing 10 ng/mL murine IL-5 (Peprotech). Media with IL-5 was changed every other day until Day 14, at which point the cells were considered mature. With each media change, the differentiating eosinophil suspension was removed and plated into a new flask, which avoided transfer of the adherent bone marrow stromal cells.

### 2.3. IL-5 Only Culture

In this protocol, eosinophils were cultured according to a published protocol by Ishihara et al. [21]. In brief, cell media was supplemented with 10 ng/mL of murine recombinant IL-5 beginning on Day 0. Media with addition of fresh IL-5 was changed every other day. Cells were considered mature from Day 7. The eosinophil suspension was transferred to a new flask with every media change.

### 2.4. Long-Lived Eosinophil (llEos) Culture

**Figure 1:**
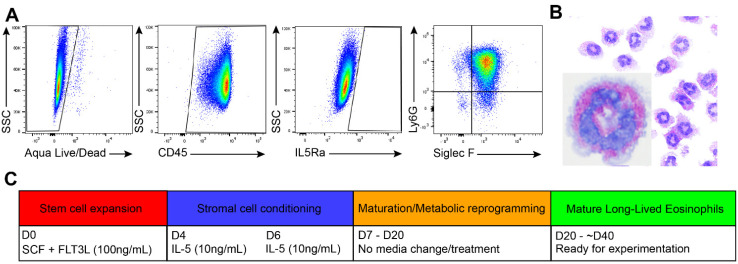
Protocol for bone marrow-derived culture of long-lived murine eosinophils (llEos). (**A**) llEos gating strategy. At maturity (culture Day 20), these cells are CD45(+), IL-5Rα(−), Siglec-F(+) and Ly6G(+). Representative flow cytometry plots are shown. (**B**) llEos are highly granulocytic, and granules stain eosin red by Diff-Quik staining. Representative image of an individual cell taken at 100× magnification. (**C**) Timeline of llEos culture. SCF = stem cell factor, FLT3L = Flt-3 ligand, IL-5 = interleukin 5.

In this newly developed protocol, results of which are reported in this article, we cultured bone marrow-derived eosinophils according to the conventional protocol (described above) with the following modifications. A total 100 ng/mL SCF and 100 ng/mL FLT3-L were added on Day 0, media was changed and supplemented with 10 ng/mL IL-5 on Days 4 and 6. Cell suspensions were transferred into a new flask with each media change. After the second addition of IL-5 (on Day 6), the cells were left untouched until Day 20 (no media change, no fresh cytokine stimulation), at which time they were found mature according to Siglec-F(+) staining by flow cytometry and characteristic differentiated eosinophil morphology (red granular staining, donut-like nuclear morphology). From Day 6, stromal cells naturally develop from stem cells when cell suspensions are allowed to settle and adhere over time, which is likely driven by autocrine programs; eosinophils are further co-cultured with stromal cells. Thus, this culture accurately represents stromal interactions experienced by eosinophils in the actual bone marrow environment. llEos differentiated using this protocol can remain viable until around Day 40, at which point viability will begin to decline. See Figure 1 for a visual schematic of this culture protocol and phenotypic appearance of these eosinophils.

### 2.5. “Old Stroma” Eosinophil Culture

This protocol is identical to the llEos culture, except that differentiating eosinophils are cultured over “old stroma”, where eosinophils are returned to their original flask with each media change. This preserves the originally precipitated stromal cells from Day 0 of culture, rather than forcing the development of a new IL-5 conditioned stromal layer after Day 6 of llEos culture (time point from which cells are left untouched in their flasks).

### 2.6. Flow Cytometry

Cell suspensions from culture were stained with Zombie Aqua Fixable Viability Kit (Biolegend, San Diego, CA, USA) for 20 min at room temperature, blocked with murine anti-CD16/CD32 (BD Biosciences, San Jose, CA, USA) for 10 min in flow staining buffer at 4 °C, and incubated with antibody cocktail in flow staining buffer for 30 min at 4 °C. The antibody cocktail contained Siglec-F (BD Biosciences; APC-Cy7), IL5Ra (BD Biosciences; Alexa Fluor 488), Ly6G (Biolegend; Alexa Fluor 700), CD45 (BD Biosciences; PE), and Lineage Antibody Mix (Ter119, CD3, CD4, CD8a, CD19, Ly6G/Ly6C, B220; Biolegend; PerCP-Cy5.5). Cells were washed in 1× PBS and fixed in 2% paraformaldehyde. Samples were run on an LSRII Flow Cytometer (BD Biosciences), and data was analyzed on FlowJo v10 software (Tree Star, Ashland, OR, USA).

### 2.7. Cytospin Preparations

Cells from culture were mounted on cytospin slides, dried, and fixed in Wright Giemsa Solution (EMD Millipore, Burlington, MA, USA). A Diff-Quik Stain Kit (Electron Microscopy Sciences, Hatfield, PA, USA) was used to differentially stain eosinophils. Eosinophils were identified by their characteristic ring-shaped nuclei and red eosin granular staining. Microscopy images were visualized on a Nikon DS-Ri2 camera microscope (Nikon, Melville, NY, USA) at 40× and 100× magnification.

### 2.8. Chemotaxis Assay

Cells were suspended in RPMI containing 1% FBS and 1% HEPES buffer at 1 × 10^6^ cells/mL. A total of 600 uL of media was added along with 50 ng/mL eotaxin to each well of a 12 well plate (no addition for controls). Cell mixture was added in 100 uL to each Transwell insert (Corning, Corning, NY, USA). Cells were incubated for two hours, after which time the inserts were removed and cells that had migrated to the lower wells were counted.

### 2.9. Fibrinogen Interaction Assay

This assay was performed according to a published protocol [22]. In brief, FITC-labeled murine fibrinogen (Oxford Biomedical Research, Rochester Hills, MI, USA) was diluted to 100 ug/mL in 1× TBS (pH 8.0, Corning). This solution was left shaking overnight at 4 °C in the wells of a 24 well plate. The following day, the solution was aspirated and blocked with FBS for 2 h at 37 °C. After, the FBS was removed and eosinophils were added to the wells at 1000 cells/uL in fresh media with 10 ng/mL IL-5 (PeproTech). The cells were left for four hours, they were then removed and the wells were fixed with 2% paraformaldehyde (ThermoFisher, Waltham, MA, USA). Plates were imaged using fluorescent confocal microscopy at 20× on an Olympus Disk Scanning Unit Confocal Microscope (phase contrast, FITC channel).

### 2.10. Biolog Metabolism Assay

Either bmEos or rEos at various time points during differentiation were seeded onto PhenoType MicroArray-Mammalian 1 or 2 (PMM1 or PMM2) Biolog Plates (Biolog, Hayward, CA, USA) in Biolog IF-M1 media. Biolog Redox Dye MB was added, and the cells were incubated in an OmniLog automated incubator/reader (Biolog) at 37 °C for 24 h. Dye reduction (measuring NAD[P]H-dependent oxidoreductase activity) was measured at 590 nm absorbance. Kinetic background values were subtracted and initial rate measurements were analyzed using Biolog Kinetic Analysis software.

### 2.11. Statistical Analysis

Statistical significance of all data was determined by t-tests or ANOVA tests followed by Tukey’s post-hoc pairwise testing, or Mann–Whitney or Kruskal–Wallis tests followed by Dunn’s multiple comparison’s test whenever data did not fit parametric test criteria. All data is represented as mean ± S.E.M. Statistical analysis was performed using GraphPad Prism 7 (GraphPad Software, Inc., La Jolla, CA, USA). An alpha level of 0.05 was used as a significance cut-off.

## 3. Results

### 3.1. Generation of Long-Lived Eosinophils (llEos) Using Modified Bone Marrow-Derived Culture Protocol

We made an unexpected discovery that aborting media and IL-5 change after Day 6 during conventional bmEos differentiation protocol results in a surprisingly healthy culture of long-lived eosinophils. According to flow cytometry, these cells were mostly CD45(+), IL5Ra(−), and double positive for Siglec-F and Ly6G on Day 20 of culture (Figure 1A). To confirm that these cells had an eosinophilic phenotype, we performed differential staining with Diff-Quik staining. These cells had “doughnut-shaped” nuclei and red staining granules, which is characteristic of a mature eosinophil (Figure 1B). For convenience, and given that these cells exhibit long-lived phenotype, we will use abbreviation “llEos” (“long-lived eosinophils”) throughout this manuscript. Eosinophils differentiated using the conventional two-week protocol will be called “bmEos” (as published previously in Dyer et al. [23]). The protocol for differentiating llEos from murine bone marrow is detailed in Figure 1C. Culture conditions resulting in llEos development suggest that changes in metabolism, as well as possible support from adherent stromal cells, are necessary for long term survival of differentiated llEos eosinophil phenotype. This also shows that once differentiated, survival of llEos is not dependent on Il-5.

### 3.2. Only the llEos Protocol Supports Long Term Survival of Differentiated Eosinophils

**Figure 2:**
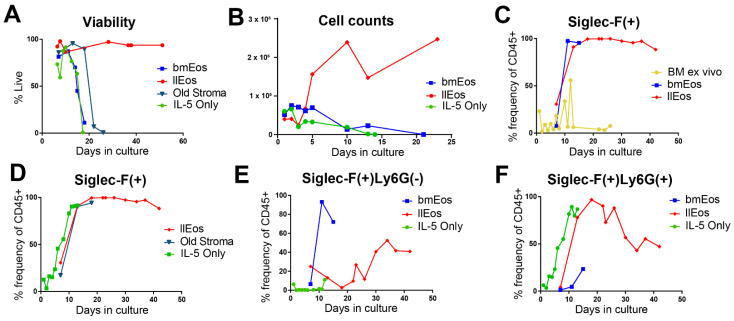
Comparison of llEos culture protocol to other eosinophil culture protocols. (**A**) Only the llEos protocol produces eosinophils capable of living past 20 days. (**B**) Unlike conventional protocols, the llEos protocol generates greater numbers of eosinophils that are sustained for the duration of the culture. (**C**) IL-5 cytokine treatment is required for bone marrow cells to differentiate the Siglec-F(+) phenotype. Untreated, homogenized bone marrow (BM ex vivo, yellow) does not spontaneously generate Siglec-F(+) despite stromal adherence; both conventional bmEos (blue) and llEos (red) eosinophil cultures do. (**D**) Other protocols, including IL-5 treatment at D0 and D4 (IL-5 only, green) and llEos without de novo stromal IL-5 conditioning (Old Stroma, navy), can produce Siglec-F(+) eosinophils. (**E**) Conventionally cultured bmEos are primarily Siglec-F(+)/Ly6G(−). Culturing with IL-5 only promotes the Siglec-F(+)Ly6G(+) phenotype. Approximately half of late-stage llEos cultures are also Siglec-F(+)Ly6G(+). (**F**) Almost all IL-5 only eosinophils and llEos in the peak of development (D20) are Siglec-F(+)Ly6G(+). Late stage llEos (D20-D40) gradually lose Ly6G expression. N = 2–4 independent experiments for each culture protocol, representative kinetic charts are shown.

To determine which factors are necessary or dispensable for the success of llEos cultures, we cultured eosinophils under a variety of conditions and measured viability, cell numbers and phenotype at the end of each protocol. We began by comparing Siglec-F expression in conventional bmEos versus llEos cultures. Both protocols resulted in nearly 100% Siglec-F(+) eosinophils out of all CD45(+) cells at maturity. This was not a spontaneous property of murine bone marrow as homogenized marrow without any cytokine stimulation or media change did not develop this phenotype (Figure 2C). We found that other culture protocols, such as culturing eosinophils with only IL-5 cytokine stimulation from Day 0, are capable of developing eosinophils with a Siglec-F(+) phenotype (Figure 2D). Similarly, culturing eosinophils over “old stroma”, where eosinophils are returned to their original flask with each media change (which preserves the originally precipitated stromal cells), resulted in Siglec-F(+) eosinophils (Figure 2D). However, none of the other protocols produced eosinophils that are as long lived and numerous as llEos (Figure 2A,B). IL-5 only and conventional bmEos cultures had a similar maximum lifespan of 14–18 days. Eosinophils cultured over old stroma were slightly longer lived, lasting around 20 days (Figure 2A). Remarkably, eosinophils in the llEos protocol survived past 40 days at nearly 100% viability (Figure 2A). In addition to having different lifespans, different culture protocols resulted in distinct eosinophil phenotypes. Conventional eosinophils were almost 100% Siglec-F(+)/Ly6G(−) at maturity (Day 14) (Figure 2E). However, both mature llEos (Day 20) and IL-5 only cultures (Day 10) were primarily Siglec-F(+)/Ly6G(+) (Figure 2F). During the later phase of their lifespan (from Day 20), llEos began to lose Ly6G expression, resulting in approximately 50% Ly6G(−) and 50% Ly6G(+) mixed population in that time frame (Figure 2E,F). In summary, this elucidates two important factors in the development of the long-lived eosinophil phenotype: (1) metabolic flexibility and (2) presence of stromal adherent cells newly derived from IL-5 primed stem cells (developed after Day 6 of culture), since return to original stroma (developed from Day 0 of culture) was not sufficient to sustain llEos phenotype.

### 3.3. llEos Exhibit Competent Chemotaxis and Promote Fibrinogenolysis

**Figure 3:**
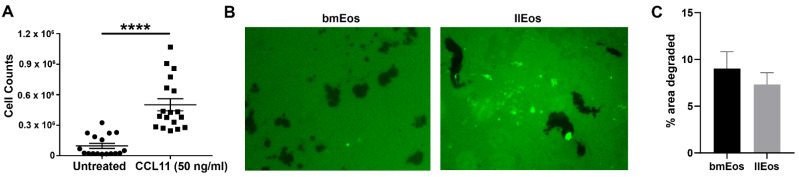
llEos can perform typical eosinophil functions. (**A**) llEos chemotax in response to eotaxin (CCL11). N = 17–19 (combined from three separate experiments). **** *p* < 0.0001 by unpaired *t*-test. (**B**) llEos degrade FITC-linked fibrinogen substrates in a similar manner to conventionally cultured bmEos. Green color shows intact FITC-linked fibrinogen. Dark spots depict areas of loss of fibrinogen substrate, visualized using confocal fluorescent microscopy. (**C**) Fibrinogen degradation quantified by pixel areas in ImageJ. Images taken at 20× magnification.

Once we confirmed that cells cultured under the llEos protocol were phenotypically similar to conventional bmEos, we performed functional tests to ensure that llEos can perform basic effector functions typical for eosinophils. Eotaxin CCL11 is a chemokine that selectively recruits eosinophils. In a chemotaxis assay, llEos exhibited significantly increased taxis to eotaxin relative to untreated controls, which was quantified by cell counts after 2 h in the assay (Figure 3A). llEos were also capable of inducing fibrinogenolysis in a similar manner to conventional eosinophils, as demonstrated by their degradation of the FITC-linked fibrinogen (Figure 3B). In summary, llEos protocol results in generation of long-lived and competent eosinophils capable of performing effector functions typical for these cells.

### 3.4. Metabolic Reprogramming Is an Important Feature of llEos Phenotype

**Figure 4:**
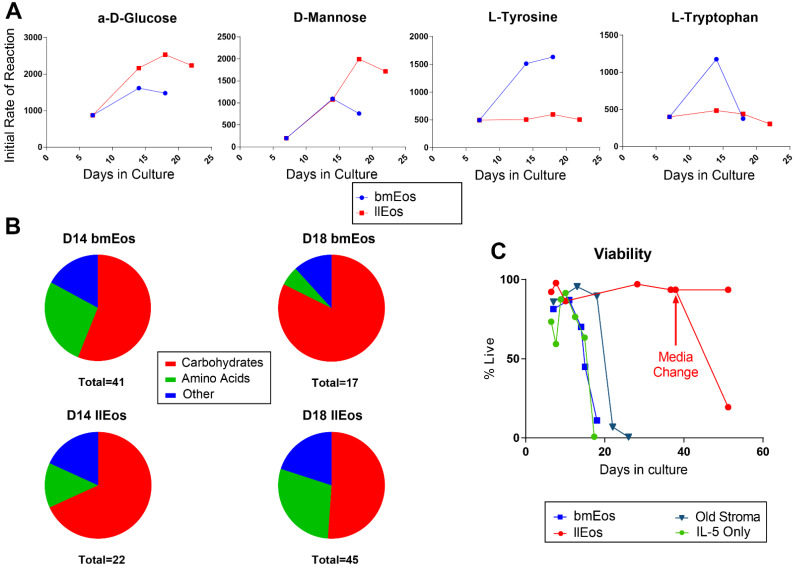
Conventional bmEos and long-lived llEos eosinophils have different metabolism. (**A**) llEos (red) preferentially metabolize certain sugars, such as α-D-Glucose and D-Mannose, as opposed to bmEos (blue), which have higher uptakes of select amino acids (tyrosine and tryptophan). (**B**) bmEos and llEos eosinophil cultures show differences in total number and diversity of preferred energy sources. Metabolic flexibility increases in llEos over time (more and higher diversity of energy substrates utilized), while it decreases in bmEos. (**C**) Eosinophils in llEos culture survive more than 40 days as long as the media conditions remain constant (nutrient-poor) and the flasks are not perturbed. If fresh media is added instead, the llEos culture declines in viability dramatically. N = 2 independent experiments, N = 1/Biolog assay, representative assay shown.

Stopping media change after Day 6 of llEos culture is one of the factors necessary to induce the long-lived eosinophil phenotype. To gain further insights into metabolic adaptations of eosinophils cultured with the llEos protocol, we performed Biolog metabolism screening assays on live cells at different time points during differentiation. In these assays, cells are presented with a variety of energy substrates, metabolism of which is quantified simultaneously by rates of NADH production in universal redox dye kinetic reactions. From screening over 150 metabolic substrates, we found that both types of eosinophils utilized different substrates at different rates over their lifespans. llEos had a propensity for increased metabolism of specific carbohydrates such as α-D-Glucose and D-Mannose, while bmEos had greater utilization of certain amino acids, including tyrosine and tryptophan (Figure 4A). To get a general sense of the overall differences in metabolic programs, we analyzed the number of substrates that each cell type utilized and categorized them by substrate type. While all eosinophils at all stages of development primarily utilized carbohydrates, there were substantial differences in the diversity of substrates used. On Day 14 of culture, bmEos metabolized 41 different substrates, while llEos metabolized only 22 substrates. Both eosinophil phenotypes favored carbohydrates as preferred energy sources. On Day 18 of culture, however, bmEos decreased diversity of metabolized substrates (only 17 detected), while llEos increased their repertoire of preferred energy sources (up to 45 from 22 on Day 14). Interestingly, by Day 18, llEos began to consume more amino acids, fatty acids and other substrates compared to bmEos, which reflects their increasing metabolic flexibility in face of continuous nutrient depletion. Due to sample size limits in these resource-demanding experiments, this data should be treated as exploratory but nevertheless helpful to illustrate the changes in metabolic processes occurring in llEos. Remarkably, if left untouched, llEos can persist for more than 40 days in culture. However, if the eosinophils are then provided with fresh nutrients in the form of a media change, llEos decline in viability almost immediately (Figure 4C). This suggests that the altered nutrient environment and metabolic reprogramming are necessary to sustain llEos for long periods of time.

### 3.5. Glucose Is Dispensable for the Maturation and Survival of llEos but Is Necessary for Adequate Expression of Siglec-F

**Figure 5:**
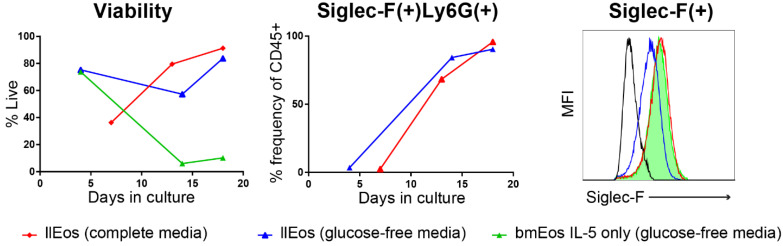
Glucose is not required for the maturation of llEos. Both llEos cultured in complete media (red) or glucose-free media (blue) are similarly viable. The ability to survive in glucose free conditions is specific to the llEos protocol, as bmEos cultured in IL-5 only protocol (green) rapidly declined in glucose-free media. However, glucose deficiency affects phenotypic development of llEos by reducing their cell surface expression of Siglec-F (measured on D13 of culture).

To further investigate this metabolic reprogramming, and specifically to examine the role of glucose in the maturation and viability of these eosinophils, we cultured llEos and bmEos in both complete media conditions and glucose-free media. We found that bmEos cultured under the IL-5 only protocol in glucose-free media rapidly decline to less than 10% of viable cells in culture (Figure 5, left). llEos remain viable in both glucose-complete and glucose-deficient media (60–85%). The viability of both these llEos cultures was similar once they reached Day 20 (approximately 80% percent). Interestingly, the majority of llEos differentiated in both glucose conditions were double positive Siglec-F(+)Ly6G(+) cells (Figure 5, middle). However, the lack of glucose in culture significantly reduced levels of Siglec-F expression on llEos by Day 13 of culture, as measured by flow cytometry (Figure 5, right). The fact that llEos can survive to maturity in glucose-free media is unique to these cells, although it does result in some phenotypic differences. This reinforces our finding that metabolic flexibility is the key to survival of long-lived eosinophils.

## 4. Discussion

In this report, we present a novel protocol of differentiating large numbers of high purity mature eosinophils with a long lifespan. With this approach, murine bone marrow progenitors are first expanded using stem cell factor (SCF) and FLT-3 ligand for four days, after which time they are treated with IL-5 two times one day apart. The key to inducing the long-lived phenotype is to stop media change during the subsequent differentiation process. This forces metabolic reprogramming and promotes long term adaptation of eosinophils to nutrient-restricted culture conditions. Another key factor is allowing stromal cells (newly derived from remaining pluripotent cells in culture) to precipitate in culture dishes during the IL-5 differentiation step. This culture system presents an improvement over past protocols, which require continuous cytokine input and only promote short term survival of mature eosinophils. Furthermore, this provides us with important insights into the ability of eosinophils to adapt to different nutrient environments and the importance of metabolism in driving eosinophil phenotypic change and survival.

Long-lived llEos display morphology typical for eosinophils. They are also functionally similar to bmEos with the ability to undergo chemotaxis in response to eotaxin, and the ability to degrade fibrinogen substrates as we previously described in detail for bmEos [22]. It is important to note that these eosinophils are reminiscent of a recently described eosinophil subset double-positive for Siglec-F and Ly6G [24,25]. In vivo, this subset of cells is prominent, although transient, in models of allergic lung inflammation, and has been shown to be induced by IL-5 treatment of bone marrow [24]. It is likely that the phenotype of these cells is related to their differentiation status. A study by Percopo et al. [25] reported that SiglecF(+)Gr1(hi) eosinophils could be detected in allergen-challenged wild-type and granule protein-deficient (EPX−/− and MBP-1−/−) mice, but not in the eosinophil-deficient ΔdblGATA strain. This suggests that this cell may be in precursor stage prior to the granular formation checkpoint (marked by expression of eosinophil proteins Epx and Mbp). Our data also shows a transient increase in the Siglec-F(+)Ly6G(+) phenotype during differentiation of llEos, which is later replaced by Siglec-F(+) only cells during the final maturation stages. Our data seems to indicate that llEos are slower to mature as opposed to bmEos, given their slow transition to the mature Siglec-F(+) phenotype (kinetics in Figure 2D,E). Interestingly, we show that IL-5 is not necessary to maintain the Siglec-F(+)Ly6G(+) phenotype in culture once it is induced. The mixed phenotype of llEos is in line with a detailed report by Limkar et al. [25] that shows that normal murine bone marrow in vivo is represented by 60% Siglec-F only and 40% Siglec-F(+)Ly6G(+) double positive eosinophils. Moreover, Limkar et al. study shows that a majority of bmEos by Day 11 of standard culture (80%) are also double positive for Siglec-F and Ly6G. Therefore, the llEos long-term protocol faithfully replicates both in vivo bone marrow eosinophils and the phenotypes of the standard eosinophil culture (used widely to study eosinophils in vitro). Although the double positive phenotype may be less mature, it is clearly a very late stage cell based on the distinct staining of red granules. It remains to be seen whether this bone marrow phenotype allows “resident” eosinophils to remain in a less activated and more flexible state to enable adaptation to changes in the local microenvironment.

Metabolism plays fascinating roles in determining the fate and survival of developing eosinophils. We show in complete, nutrient-rich culture conditions that bmEos decrease the diversity of their preferred energy sources (from 41 on Day 14 of culture to 17 on Day 18 of culture), which suggests that eosinophils become more metabolically restricted as they mature. On the contrary, our data shows that the metabolic flexibility of llEos increases over time (from 22 preferred energy sources on Day 14 of culture to 45 on Day 18 of culture) as they are forced to differentiate in a media-depleted, nutritionally-restricted environment. The metabolic differences between bmEos and llEos could also be explained by temporal differences in maturation kinetics and the general decline of bmEos after Day 14 of culture. However, we believe that metabolism differences are primarily driven by a combination of ongoing differentiation and suppression of nutrients in cell media of llEos (stopping media change), which forces these cells to adapt their metabolism for survival before they mature. As a general pattern, slower metabolisms are associated with longer lifespans [26], which may help explain why llEos have such substantially prolonged lifespans. Interestingly, llEos increase their preference for amino acid metabolism over time, while bmEos begin to metabolize a greater number of carbohydrates. Surprisingly, switching fully developed llEos back to nutrient-rich media immediately impacted their survival, suggesting a “reprogrammed” phenotype tuned to current culture conditions. Altogether, our data seems to indicate that flexible metabolism induced by nutritional challenge during the differentiation process is what promotes the long term survival of eosinophils and adaptation to their immediate microenvironment. The research area of eosinophil metabolism is currently in its infancy [27,28]. We do not know yet whether currently known subsets (or activation states) of tissue eosinophils are characterized or determined by their metabolism. In support of the existence of metabolic differences between eosinophil subsets, a study by Andreev et al. [29] found that rEos versus iEos sorted from bone marrow and blood of IL-5 transgenic mice exhibit differences in metabolism (glycolysis, basal respiration and ATP production). The importance of understanding the relationship between metabolism and immune phenotype is exemplified by research of other cell types, such as macrophages. For example, we know now that alternative activation and immune profile of tissue macrophages are driven by changes in cellular metabolism [30,31].

In our culture conditions, it appears that another key determinant of eosinophil longevity lies in the stromal layer. In conventional bmEos protocols, eosinophils are deprived of stromal interactions by being transferred to new flasks with each media change. In llEos culture, eosinophils are allowed to interact with newly adherent stroma from Day 7 of culture. Interestingly, when llEos were cultured under the same conditions but being constantly re-exposed to the old stromal layer, rather than being replated into new flasks, the resulting eosinophils did not live as long (Figure 2). This implies that the cytokine regimen affects not only the eosinophils but also the developing stromal cells (newly precipitated from pluripotent cells during IL-5 treatment of culture, Days 6–8), and that these two cell populations become programmed in parallel to be able to survive long term after initial IL-5 conditioning. Interestingly, once set on the differentiation course, IL-5 becomes completely dispensable for the reminder of culture time course. It is possible that the stromal layer produces a particular growth and survival factor driving eosinophil survival. GM-CSF is a very likely candidate, as it has been shown to be produced by stromal cells and promote long-term eosinophils [32,33]. It is also possible that llEos produce their own GM-CSF as an autocrine survival factor, which has been reported for human eosinophils cultured with ECM [34] and in asthmatic airway [35]. This is one of the possible mechanisms for IL-5 independent survival of tissue eosinophils in asthma and eosinophilic esophagitis patients treated with anti-IL-5 biologicals, which only deplete about 50–60% of tissue eosinophils. ECM constituents could be other likely candidates. Our previous research showed that ECM protein tenascin-C, which is typically induced in tissues experiencing repair and remodeling, suppresses differentiation but enhances viability of eosinophil precursors [15].

More research is necessary to determine the key aspects of metabolism and stromal-eosinophil interactions necessary or sufficient to drive long-lived IL-5-independent phenotypes. Gaining a better understanding of this interaction could greatly advance our future understanding of tissue eosinophils and reasons for successes and failures of today’s biological therapies.

## Data Availability

The study did not report any data for repository upload.
